# The aesthetic nature of the birthing room environment may alter the need for obstetrical interventions *– an observational retrospective cohort study*

**DOI:** 10.1038/s41598-018-36416-x

**Published:** 2019-01-22

**Authors:** Tine Wrønding, Aikaterini Argyraki, Jesper Friis Petersen, Märta Fink Topsøe, Paul Michael Petersen, Ellen C. L. Løkkegaard

**Affiliations:** 1 Department of Gynecology and Obstetrics, North Zealand Hospital, Hillerød, University of Copenhagen, Copenhagen, Denmark; 2 Department of Photonics Engineering, Technical University of Denmark (DTU), Frederiksborgvej 399, DK, 4000 Roskilde, Denmark

## Abstract

The concept of sensory delivery rooms was introduced in 2013. These rooms offer programmable calming lights, restful blurred pictures displayed on a wall-sized big screen, and sound effects. The primary aim of this observational study was to analyse the risk of obstetrical interventions among women giving birth for the first-time in a sensory delivery room vs. a standard delivery room. We included nulliparous, term pregnant women having a single baby with a cephalic presentation who were in spontaneous labour and gave birth between March 1^st^ 2014 and July 1^st^ 2015 in North Zealand Hospital, Hillerød. A total of 789 women were included in the study, 313 gave birth in a sensory room and 476 in a standard delivery room. The risk of a caesarean delivery was significantly decreased when giving birth in a sensory room compared with a standard delivery room (OR, multiple adjusted: 0.44; 95% CI 0.22–0.87); furthermore, the use of oxytocin infusion was also reduced (OR, multiple adjusted: 0.71; 95% CI 0.50–1.03). This observational cohort study suggests that giving birth in a sensory delivery room could lower the risk of caesarean delivery, potentially reducing the number of such deliveries by one for every 23 patients.

## Introduction

**Figure 1:**
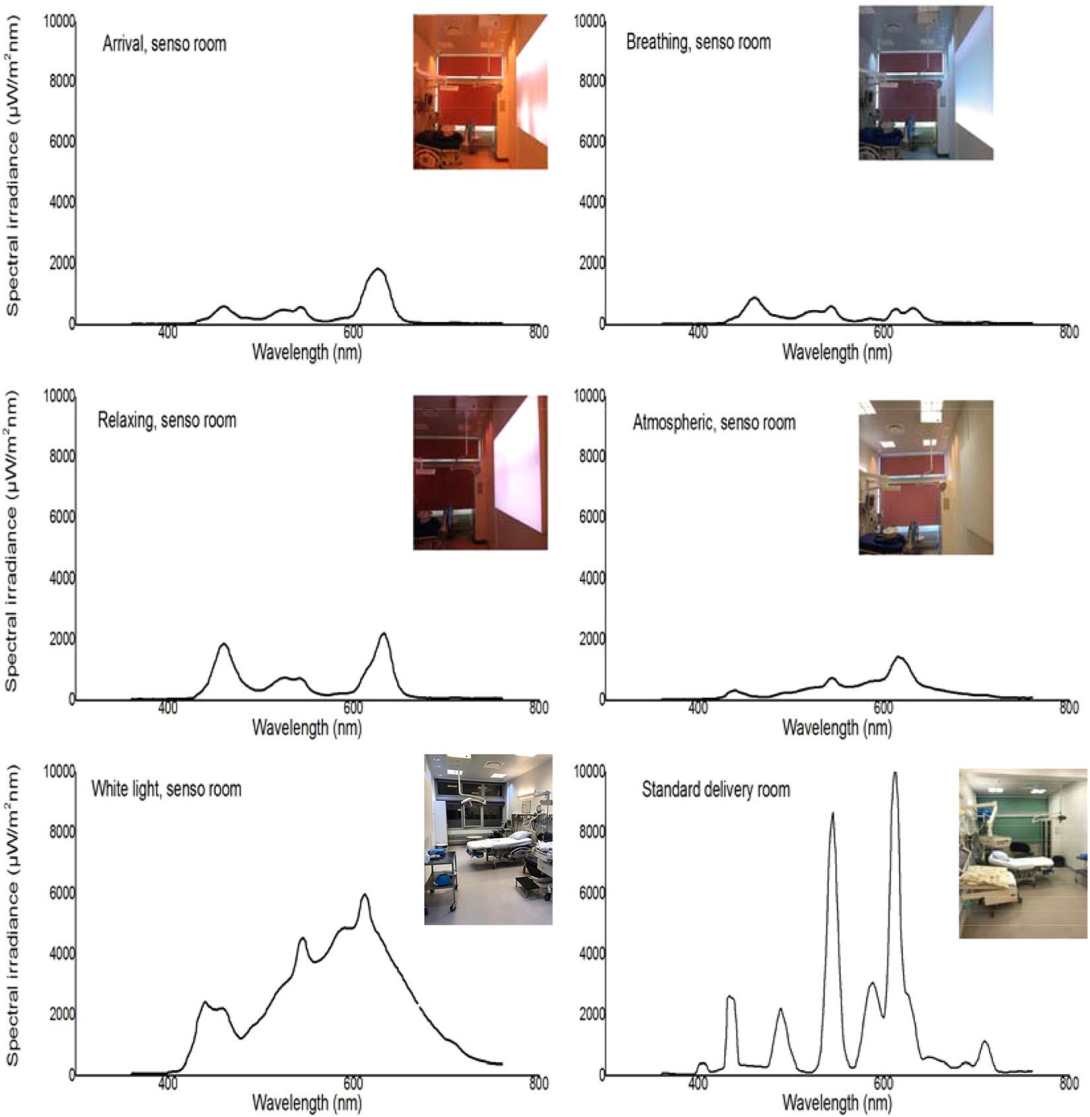
The various light programs and spectral irradiances in a sensory delivery room and standard delivery room. The average irradiance and illuminance level in the standard delivery room is much higher than in the sensory delivery rooms.

The concept of sensory delivery rooms was introduced in Denmark in 2013 for women in labour. The sensory delivery rooms offer programmable calming lights (Fig. 1) with low irradiance, restful blurred pictures displayed on a wall-sized big screen, and sound effects. Most primary births commence at night or in the early morning hours^1^ when it is dark or when the light has less significant blue wavelength contributions. Thus, alteration of light could influence labour progression and outcome.

## Aim

The aim of this study was to analyse the risk of obstetrical interventions and complications among nulliparous women, in spontaneous labour at term with one foetus in cephalic presentation (Robson 1^2^), in sensory delivery rooms with spectral light settings compared with the light settings in the standard delivery rooms. The primary outcomes were the use of additional oxytocin during labour and the risk of caesarean delivery. Secondary outcomes comprised the need for vacuum extraction, need for an episiotomy, rate of rupture of the anal sphincter, length of birth, parturition period, the rate of postpartum haemorrhage and neonatal outcome.

## Results

**Figure 2:**
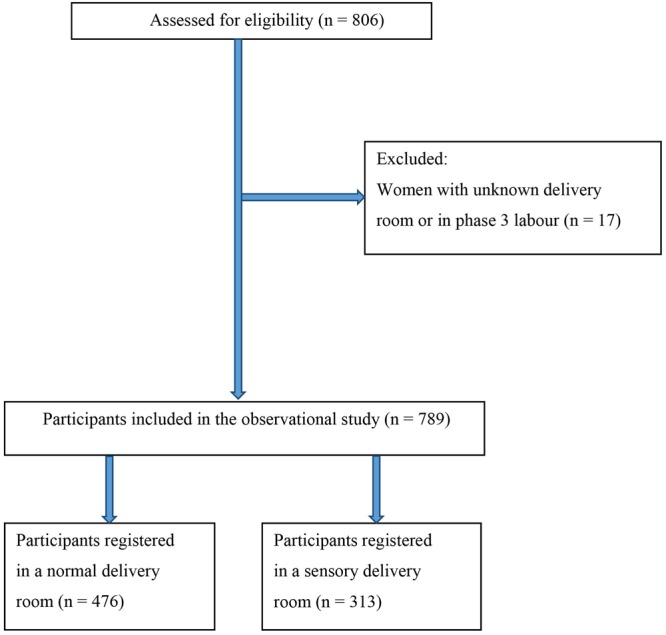
Flowchart for the observational cohort.

Initially, 806 records were screened for eligibility; of these, 17 were excluded as the type of delivery room was not recorded or the women giving birth were fully dilated, in the active stage, and pushing when arriving to the hospital (Fig. 2). A total of 789 cases were included in the study, 313 gave birth in the sensory delivery room and 476 in the standard delivery room. In the overall study period, 3,201 women gave birth at North Zealand Hospital, Hillerød; thus, 25.2% were included in this study. The baseline characteristics of the women giving birth in the sensory and the standard delivery room groups are summarized in Table 1. No statistically significant differences were observed between the two groups. The caesarean delivery rate was significantly lower in the group of women giving birth in a sensory delivery room (6.4%) compared with the group giving birth in a standard delivery room (10.7%); (OR 0.57; 95% CI 0.33–0.97) (Table 2). Multiple adjustments for the potential confounders maternal age, use of oxytocin infusion during birth, epidural analgesia, gestational age, weight of the child, degree of cervical dilatation when admitted to a labour room and meconium-stained amniotic fluid did not change the result (OR 0.44; 95% CI 0.22–0.87) (Table 2). Based on these results, the calculated number needed to treat in a sensory delivery room to avoid one caesarean delivery was 23. Indication for caesarean section was asphyxia (standard: 21%, sensory: 33%), inefficient uterine action (standard: 21%, sensory: 28%), cephalopelvic disproportion (standard 47%, sensory: 39%), failed vacuum extraction (standard: 9%, sensory: 0%) and maternal request (standard: 2%, sensory: 0%). The univariate analysis indicated no association between the use of additional oxytocin infusion and the type of delivery room (OR 0.83; 95% CI 0.61–1.13). However, when adjusting for the potential confounders maternal age, epidural analgesia, gestational age, weight of the child, and meconium-stained amniotic fluid and degree of cervical dilatation when admitted to a labour room, the estimated risk for this outcome nearly reached statistical significance (OR 0.71; 95% CI 0.50–1.03) (Table 2).

**Table 1:** Characteristics of participants.

Characteristics	Delivery room	
	Standard n = 476	Sensory n = 313
Age (years), mean ± SD (range)	27.7 ± 5.1 (16–43)	28.5 ± 4.9 (17–42)
Gestational age, mean ± SD (range)	40.02 ± 0.96 (37–42)	40.05 ± 0.99 (37–42)
Comorbidity, n (%)	22 (4.6%)	14 (4.5%)
Epidural, n (%)	180 (37.8%)	119 (38.0%)
Weight of child (grams), mean ± SD (range)	3.464 ± 406 (2.390–4.820))	3.512 ± 435 (2,330–4.960)
Degree of cervical dilatation when admitted to a labour room, mean ± SD (range)	4.1 ± 2.2 (0–10)	4.4 ± 2.4 (0–10)
Degree of cervical dilatation at section mean ± SD (range), n	8.2 ± 1.7 (4–10) n = 51	8.6 ± 1.9 (4–10) n = 20

**Table 2:** Odds ratios (OR) of risks in the sensory delivery room vs. standard delivery room.

PRIMARY OUTCOMES	Standard n (%)	Sensory n (%)	OR	95% CI	OR adjusted^a^	95% CI	OR multi-adjusted^b^	95% CI
Caesarean Delivery	51 (10.7%)	20 (6.4%)	0.57	0.33–0.97	0.54	0.31–0.92	0.44	0.23–0.87
Oxytocin infusion	164 (34.5%)	94 (30.0%)	0.83	0.61–1.13	0.77	0.56–1.06	0.71	0.50–1.03
SECONDARY OUTCOMES								
Vacuum extraction	57 (12%)	32 (10.2%)	0.45	0.53–1.32	0.79	0.49–1.25	0.86	0.52–1.43
Episiotomy	25 (5.3%)	17 (5.4%)	1.04	0.55–1.95	1.00	0.53–1.80	1.15	0.59–2.25
Anal sphincter rupture	11 (2.3%)	5 (1.6%)	0.69	0.24–1.99	0.65	0.22–1.89	0.64	0.20–2.02
Meconium stained liquor	47 (9.9%)	20 (6.4%)	0.62	0.36–1.07	0.59	0.34–1.04	0.58	0.33–1.02
Postpartum Haemorrhage > 500 ml	103 (21.6%)	69 (22%)	1.02	0.72–1.44	1.06	0.73–1.51	1.02	0.69–1.50
Postpartum Haemorrhage > 1000 ml	22 (4.6%)	24 (7.7%)	1.71	0.94–3.11	1.78	0.96–3.27	2.01	1.07–4.09
Apgar 1 min < 7	16 (3.4%)	15 (4.8%)	1.45	0.70–2.97	1.47	0.71–3.06	1.55	0.71–3.38
Apgar 5 min < 7	1 (0.2%)	1 (0.3%)	1.52	0.10–24.43	1.43	0.10–23.60	NA	NA
A. Umbilical cord < 7.10	24 (5.6%)	12 (4.1%)	0.72	0.35–1.46	0.70	0.34–1.42	0.65	0.29–1.45

^a^Maternal age, use of oxytocin infusion during birth, and epidural analgesia.

^b^Maternal age, use of oxytocin infusion during birth, epidural analgesia, meconium-stained liquor (not included in the model asssing risk of meconium-stained liquor as outcome), gestational age, degree of cervical dilatation when admitted to a labour room and weight of the child.

OR Odds Ratio.

NA Not Assessed.

**Figure 3:**
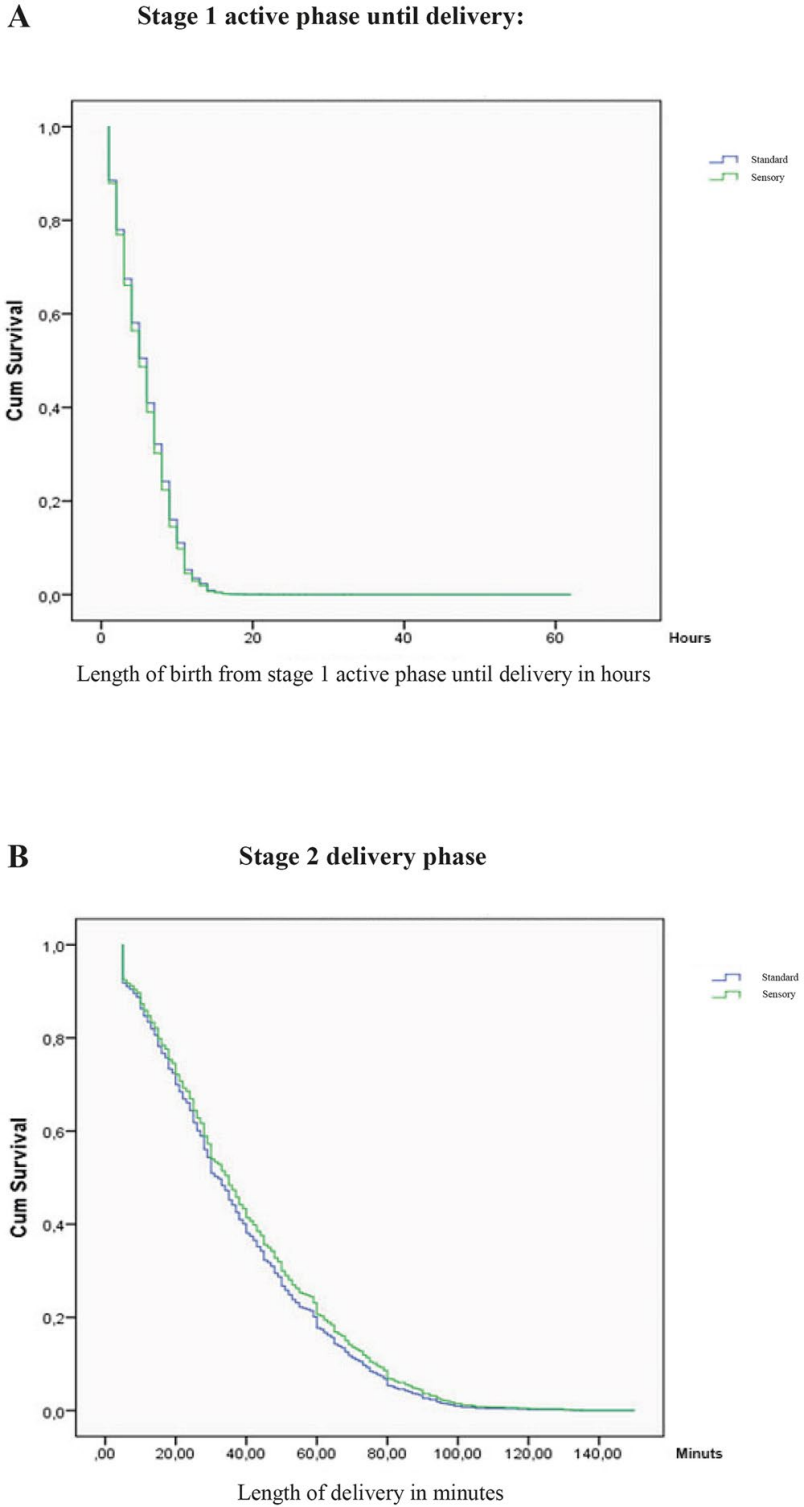
(**A**) Kaplan-Meier curve of the duration of labour in the active phase until birth of the child. (**B**) Kaplan-Meier curve of the parturition phase (stage 2 delivery phase) in sensory and standard delivery rooms.

There was increased risk of severe postpartum haemorrhage in the multiple adjusted model in the sensory compared to standard delivery room and no statistically significant difference between the two groups regarding the other secondary outcomes of episiotomy, use of vacuum extraction, meconium-stained liquor, neonatal outcome assessed by Apgar after 1 minute or 5 minutes or arterial pH values, and rupture of the anal sphincter (Table 2). There was also no statistically significant difference found for the overall length of birth, also after adjustment for the potential confounders of maternal age, epidural analgesia, gestational age, weight of the child, and meconium-stained amniotic fluid and degree of cervical dilatation when admitted to a labour room (stage 1–3) (HR 0.95; 95% CI 0.82–1.11). The interquartile range of the active phase in stage 1 until birth of the child in the sensory delivery room group was 7 h vs. 6 h for the standard delivery room. Regarding the time for delivery (when the woman is pushing), the two groups did not significantly differ, with an average of 35 min in the sensory delivery room and 37 min in the standard delivery room, multiple adjusted, (HR 1.01; 95% CI 0.47–1.26) (Fig. 3A,B).

## Discussion

We found a significantly reduced risk of caesarean delivery for women giving birth in a sensory delivery room compared with a standard delivery room. Furthermore, the need for oxytocin infusion was lower in the group giving birth in a sensory delivery room vs. a standard delivery room. The frequency of having an acute caesarean delivery for the Robson 1 group was 9.7% in Denmark between 2009–2011^3^. The caesarean delivery rate among the Robson 1 group in this period at the Department of Obstetrics and Gynaecology at Northern Zealand Hospital Hillerød was 9.0%. In our study group, the caesarean delivery rate was 10.7% in the standard delivery room. When evaluating stimulation with oxytocin during birth, our univariate analysis suggested equal use among women giving birth in the two types of delivery rooms (30.4% in sensory delivery rooms vs. 34.5% in standard delivery rooms). These unadjusted percentages are comparable to the average use of oxytocin stimulation during birth in hospitals in Denmark^4^. However, when adjusted for potential important confounders, the estimated risk for this outcome nearly reached statistical significance. We found the two groups, giving birth in either the sensory or the standard delivery room, were comparable as no difference was observed between baseline characteristics. In only one case, a woman changed from a standard to a sensory delivery room during labour when it became available. She was recorded as giving birth in a standard delivery room. To our knowledge, this study is the first to highlight the potential positive effects of giving birth in a sensory delivery room.

A potential weakness of the study is its observational nature. To some extent, the women in labour were assigned by midwifes to either a sensory delivery room or a standard delivery room. The midwives could potentially select women who were more likely to have an uncomplicated birth to the sensory rooms since they were more likely to enjoy the full potential of the room, and women with more complicated labour to the standard delivery room. This would be a potential selection bias. To explore this potential bias, 10 midwifes of approximately 60 on the labour ward at the Department of Obstetrics and Gynaecology at North Zealand Hospital, Hillerød, were randomly asked if they selected or had any preferences for women to give birth in either of the two types of delivery rooms. All midwifes answered that they did not make any selection, and that the two sensory rooms (of the 10 delivery rooms) at the hospital were the first to be chosen either by the woman in labour or the midwife; thus, they were consequently used more often. Additionally, we only included nulliparous women with fetuses in the cephalic position who were in spontaneous labour at term (Robson 1^2^) in the study. The systemic classification into a Robson group^2^ by midwifes or doctors was not done when the pregnant woman arrived at the hospital in labour. This supports the notion of a random selection of women to the sensory delivery rooms.

In the present study we do not have any information on Body Mass Index (BMI) at the time of labour. Hereby, we cannot guarantee that the two groups are equally divided concerning BMI. It is well-documented that obese women have a higher risk of acute caesarean delivery and a lower probability for a normal vaginal delivery^5^. Studies suggest most primiparous births commence at night or in the early morning hours^1^. In the morning, melatonin excretion is maximal. Melatonin is a hormone^6^ produced in the pineal gland that has a 24-h secretory rhythm. This rhythm is driven by an endogenous circadian oscillator in the suprachiasmatic nucleus (SCN) of the hypothalamus^7^. A neuronal pathway connects the SCN with spinal nuclei of sympathetic neurons that activate the pineal gland. The neuronal connection from the retina to SCN (via the retinohypothalamic tract) imparts the suppressive effect of the light and the involvement of the melatonin secretory rhythm on the light-dark cycle^8^. Oxytocin is produced in the hypothalamus and excreted from the posterior pituitary gland. Oxytocin is the most important hormone in initiation of labour and acts synergistically with melatonin^9^. Like oxytocin, melatonin has its own receptors on the uterus capable of providing contractions. These receptors interact with each other and, along with additional hormones, induce contractions of the uterus^9^. There are several published studies on circadian rhythm and initiations of births^9^. Light that inhibits melatonin secretion is blue (446–477 nm) or white at high illuminance (around 10,000 lx) and red/yellow light (625–740 nm) is believed to have a non-suppression effect on melatonin^10,11^.

Labour is traditionally divided into three stages. In labour, and several hormones play essential roles. These hormones includes prostaglandin, oxytocin, melatonin, and adrenaline^10^. Studies suggest that adrenaline has a prolonging effect in stage 1 of birth^11^. Oxytocin, prostaglandin, and melatonin are important hormones regarding for uterine contractions and are therefore essential in all three stages^12^.

In 2014, Olcede J. *et al*.^13,14^ published a pilot study on pregnant volunteers (>38 weeks of gestation) and performed continuously monitoring for uterine contractions from 7:00 PM until 7:00 AM under dim light. At 11:00 PM, a 10,000 lux full- spectrum lamp, one meter from the participants’ eyes was activated for one hour to suppress melatonin secretion^15^. Among the volunteers exposed to light, the nocturnal contraction frequencies were either partially or completely suppressed. This observation supports the idea that light levels that potentially could facilitate the secretion of melatonin. A continuous secretion of melatonin during birth could induce continuous contractions of the uterus, and hereby optimal propulsion for the women in labour. In our study, women who give birth in a sensory delivery room, theoretically would have the best conditions for a continuously secretion of melatonin and, therefore, the opportunity for optimal propulsion. Yet, we did not see any difference in the time length of birth in the two groups. This could be due to the fact because we did not record which light program was chosen, and for how long the sensory programs were applied during labour. Moreover, if the sensory programs were shut down for a period of time, or what time of day the birth took place - if it was night or day. Also, several other parameters influence the propulsion of the birth. Siw Alehagen *et al*.^11^ stated that fear and pain negatively correlated with the duration of the early phases in birth have a negative impact on the early phases of birth due to a rise in levels of epinephrine which are negatively correlated with the duration of the early phases in birth^15^.

## Materials and Methods

In August 2013, a local project of innovation was the establishment of sensory delivery rooms that included certain light settings. This was developed in corporation with Philips and Wavecare in two of the ten delivery rooms at the labour ward at North Zealand Hospital, Hillerød, Denmark. In these sensory delivery rooms, there were five optional sensory programs with different auditory and visual stimulation regarding colored lightning and soft soundtracks. The visuals were displayed on a large screen as blurred dynamic light that was reflected on the walls. The lightning and screen pictures could shift colours: blue, green, yellow, red, and white. The five pre-set sensory programs included: Arrival (red), relaxation (red-blue), breathing (blue), atmospheric (red-yellow), and white light with no sound. The spectral irradiances of the different light pre-settings in the sensory room, as well as the spectral irradiance of the standard delivery room, are presented in Fig. 1. The light characterization measurements were performed on location with a handheld spectrometer from UPRtek (MK 350, Zhunan, Taiwan). In these pre-set sensory programs, there was also an option to slightly change the wavelength distribution among the available colours. The average illuminance level in the standard delivery room (260 lx) is much higher than in sensory delivery rooms (83 lx).

## Methods

We established a retrospective cohort and collected data from medical records from March 1^st^ 2014 to July 1^st^ 2015 at North Zealand Hospital, Hillerød, Denmark. We included Robson classification birth group 1^2^, which comprises term (37 + 0–42 + 0) nulliparous women, with a single foetus in the head position, in spontaneous labour^3^. Upon arrival at the delivery ward, the women in labour were assigned to a sensory delivery room or a standard delivery room based on availability and preferences. The midwives recorded the type of delivery room in the medical record as well as registered the information in a local protocol kept on the labour ward. All data used in this study were collected from the chart and recorded in an Access database. Data regarding age, gestational age determined by ultrasound, comorbidities (gestational diabetes mellitus, depression, hypertension, other chronic diseases, or psychological diseases), epidural analgesia, meconium-stained amniotic fluid, degree of cervical dilatation when admitted to a labour room, degree of cervical dilatation at section and the weight of the foetus were recorded and included as confounders in multiple analyses. The age of the mother in years (<24, 25–29, 30–34, and >35), gestational age in weeks (37–39, 40, 41, and 42), and weight of the fetus in grams (<2,900; 3,000–3,400; 3,500–3,900 and >4,000) were divided into categories and included as categorical variables. Information on postpartum haemorrhage (measured as total amount of blood loss in mL) and categorized in two variables divided at 500 ml and 1000 ml was collected. Furthermore, neonatal outcome as APGAR score after 1 minute and 5 minute and blood-pH from the arteria of the umbilical cord was collected.

The identification and definition of asphyxia, inefficient uterine action and cephalopelvic disproportion as indication for caesarean section was based upon local guidelines. Asphyxia was suspected by auscultation, CTG and/or thick green meconium-stained amniotic fluid. In case of Asphyxia suspicion a scalp-pH from the fetus was analyzed. Depending on the value the birth could continue with CTG monitoring (pH > 7.25) or with repeated scalp-pH monitoring (pH 7.20–7.25) or acute delivery either with vacuum extraction if possible or with cesarean section (pH < 7.20).

Inefficient uterine action was diagnosed in the active phase when the orificium has dilated less than 2 cm evaluated over 4 hours, after fully dilation when it was estimated that the caput would reach the pelvic floor in 3 hours, and during the fetal expulsion phase when it was estimated the child would not be born after no later than 2 hours.

Cephalopelvic disproportion indicates a mismatch between the fetus’s head and the size of the womans pelvis, possible due to small pelvis, abnormal pelvic (caused by illness or trauma) or large fetus (caused by diabetes, genetically large fetus or past due date). The suspicion of cephalopelvic disproportion will occur due to slow progression during birth despite satisfying contractions and/or despite oxytocin infusion. It can be difficult to diagnose whether there is a cephalopelvic disproportion or dystocia. In Denmark, dystocia is generally used for both slowly and lacking progress in birth. During acute caesarean section, it will be clinically determined whether there was an inappropriate presentation of the fetus head or cephalopelvic disproportion.

Data on the predefined primary and secondary outcomes were collected from medical records. Primary outcomes were the use of oxytocin during birth and caesarean delivery. Secondary outcome comprised vacuum extraction, meconium-stained liquor, neonatal outcome, episiotomy, rupture of the anal sphincter, and length of birth categorized into stages. Labour is traditionally divided into three stages. Stage 1 consists of early labour, active labour, and transition; stage 2 is the phase from a fully dilated cervix until delivery divided in a passive and an active stage; and stage 3 is the birth of the placenta. All stages were measured in minutes. The study was approved by The Danish Health and Medicine Authority (case j.nr. 3–3013–899/1) and The Danish Data Protection Agency (NOH-2014-042 I-suite nr.: 03413).

### Statistical methods

Initial descriptive univariate analyses were conducted on the baseline characteristics of the two groups of women giving birth in either the sensory or the standard delivery rooms. Student’s t-Test, Chi-square Test, and interquartile ranges were applied for continuous and category dichotomous outcomes, respectively. Logistic regression was fitted to estimate the odds ratios in univariate and multiple logistic regression analyses for the primary and secondary outcomes. Furthermore, Kaplan-Meier survival curves were fitted for the length of birth for the Hazard Ratio. In addition, the unadjusted number needed to treat was calculated.

## Conclusion

This observational cohort study suggests that giving birth in a sensory delivery room could be associated with a lower risk of caesarean delivery, which could have great clinical importance; however, randomised clinical trials are needed to verify this finding.

## Data Availability

The datasets generated during and/or analysed during the current study are available from the corresponding author on reasonable request. We confirm that all methods were carried out in accordance with relevant guidelines and regulations. This study dose not contain any experiments on humans and/or the use of human tissue samples. Informed consent was giving throw The Danish Health and Medicine Authority and The Danish Data Protection Agency- see below. The study was approved by The Danish Health and Medicine Authority (case j.nr. 3-3013-899/1) and The Danish Data Protection Agency (NOH-2014-042 I-suite nr.: 03413).
